# Decompensated Non-Ischemic Cardiomyopathy Induced by Anabolic-Androgenic Steroid Abuse

**DOI:** 10.7759/cureus.11476

**Published:** 2020-11-13

**Authors:** Palwinder Sodhi, Meera R Patel, Anup Solsi, Pallavi Bellamkonda

**Affiliations:** 1 Cardiology, Creighton University School of Medicine, St. Joseph's Hospital and Medical Center, Phoenix, USA; 2 Internal Medicine, Creighton University School of Medicine, St. Joseph's Hospital and Medical Center, Phoenix, USA; 3 Cardiovascular Disease, Creighton University School of Medicine, St. Joseph's Hospital and Medical Center, Phoenix, USA

**Keywords:** dilated cardiomyopathy, anabolic androgenic steroid, systolic heart failure

## Abstract

A 30-year-old male presented to the emergency department with dyspnea, fatigue, orthopnea, and paroxysmal nocturnal dyspnea for the past three months. The patient admitted to anabolic steroid use for the past 11 years. Transthoracic echocardiography was significant for severely dilated left ventricle, diffuse hypokinesis, ejection fraction < 15%, and grade II diastolic dysfunction. The patient was diagnosed with decompensated, non-ischemic cardiomyopathy stage C, and New York Heart Classification (NYHA) class III > IV, likely from use of anabolic steroids, after a negative workup for other etiologies. On follow-up after continuation of guideline-directed medical therapy, the patient demonstrated improved heart failure status (NYHA class I > II). Cardiomyopathy is a rare but important adverse effect of anabolic steroids to consider.

## Introduction

Since the 1980s, the male sex hormone testosterone and its artificially derived forms, collectively known as anabolic-androgenic steroids (AASs), have been used illicitly by millions of males and females alike as a way to enhance muscle mass. Commonly misused drugs include stanozolol, methandrostenolone, nandrolone, and androstenedione. AASs are currently approved to treat a variety of medical conditions such as male hypogonadism, anemia due to renal and bone marrow failure, and HIV wasting syndrome; however, they continue to be abused for non-medical reasons [1]. As more people have been seen taking these substances, medical literature has documented the extensive adverse effects of these medications, including renal and hepatic dysfunction, hematological abnormalities (polycythemia), metabolic and reproductive disorders, psychiatric disturbances, and, most notably, cardiovascular (CVD) disease [2,3]. Prior studies have demonstrated an association between AAS and CVD in the form of stimulating cardiac myocyte hypertrophy, lowering arrhythmia threshold, increasing risk of atherosclerosis, and causing myocardial left ventricular dysfunction leading to cardiomyopathy and even sudden cardiac death [4]. Although adverse CVD effects have been seen with AAS, the relationship is incompletely understood [5]. In this report, we present a case of a 30-year-old male who developed acute decompensated heart failure from long-term anabolic steroid use.

## Case presentation

**Figure 1:**
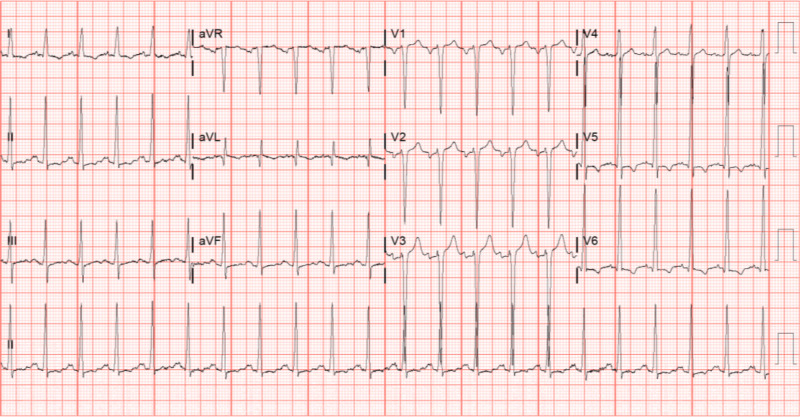
ECG illustrating sinus tachycardia at the rate of 132 beats per minute. The S wave in V1 + R wave in either V5 or V6 totals >35 mm, meeting the Sokolow-Lyon ECG criteria for LVH. ECG, electrocardiogram; LVH, left ventricular hypertrophy

A 30-year-old Caucasian male with no significant medical history presented to the emergency department with complaints of worsening dyspnea, increased exertional fatigue, orthopnea, and paroxysmal nocturnal dyspnea for the last three months, with acute worsening over the last four to five days. He reported remote tobacco (unable to quantify pack-years), marijuana, and cocaine (intermittent, years ago) use along with intermittent use of anabolic steroids over the last 11 years. The patient is currently on testosterone cypionate after recently finishing a cycle of trenbolone acetate. The patient reported that he had completed multiple cycles of anabolic steroids over the years. He was seen at an urgent care two days prior to admission and was given Flonase and albuterol with no relief of symptoms. On admission, BMI was 29.02 kg/m^2^, vitals were significant for tachycardia into the 130s and tachypnea. Cardiopulmonary examination revealed jugular venous distension and crackles in bilateral lower lung fields. Laboratory investigations were notable for a hemoglobin of 17.5 gm/dL, sodium of 141 mmol/L, potassium of 4.0 mmol/L, creatinine of 1.17 mg/dL, bicarbonate of 24 mmol/L, D-dimer of 2,401 ng/mL, and B-natriuretic peptide of 354.4 pg/mL. Urine drug screen was negative. Troponin I was mildly elevated at 0.029. Electrocardiogram (ECG) showed sinus tachycardia with left ventricular hypertrophy (Figure 1).

**Figure 2:**
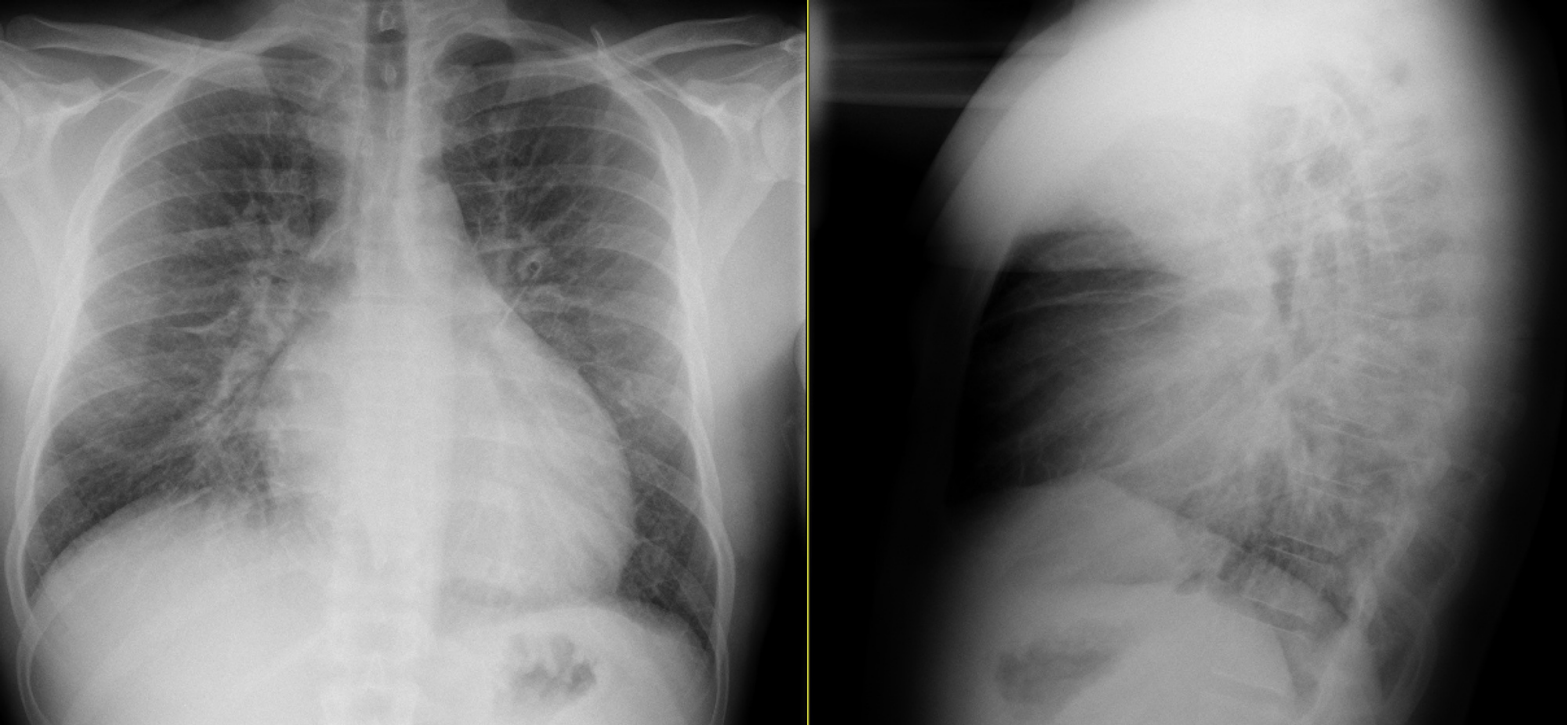
CXR showing prominent interstitial opacifications concerning for an infectious process versus pulmonary edema. Based on the lateral view, cardiac dimensions appeared within normal limits. No findings of obstructive lung disease were noted. CXR, chest X-ray

**Figure 3:**
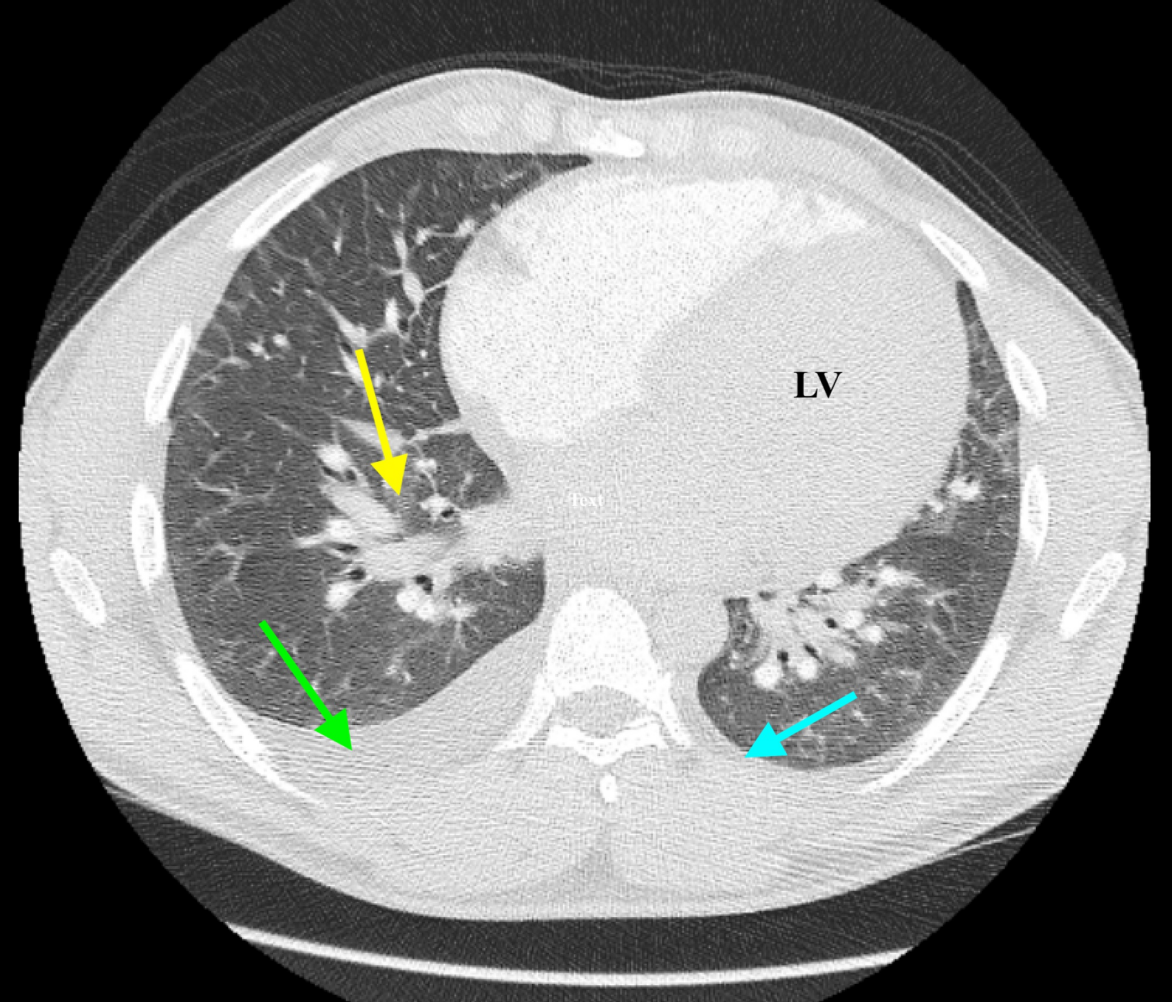
CTA of the chest demonstrating mild-to-moderate R pleural effusion (green arrow), small left pleural effusion (blue arrow), pulmonary vascular congestion consistent with pulmonary edema (yellow arrow), and enlarged heart with dilated LV. Obstructive lung disease changes were not visualized. CTA, computed tomography angiography; LV, left ventricle

Chest X-ray depicted prominent interstitial opacifications and no evidence of obstructive lung disease (Figure 2). Computed tomography angiography (CTA) of the chest was obtained, which did not demonstrate a pulmonary embolism but showed an enlarged left ventricle (LV), small right-sided pleural effusion, and evidence of likely cardiogenic pulmonary edema (Figure 3).

 

Transthoracic echocardiography (TTE) was significant for a severely dilated LV, diffuse hypokinesis, ejection fraction (EF) < 15%, and grade II diastolic dysfunction. LV end-systolic dimension and end-diastolic dimensions were measured at 7.5 cm and 7.7 cm, respectively (reference ranges: 2.5-4.0 cm and 4.2-5.8 cm, respectively). The left atrium (LA) was mildly dilated at 4.2 cm (normal: 3.0-4.0 cm). LV wall thickness was normal. The right ventricle (RV) had normal size and systolic function. The right atrium was within normal size limits. No significant valvular abnormalities were noted. RV systolic pressure was measured at 58 mmHg (Videos 1-3). There was no myocardial strain imaging performed. No prior TTE studies were available for comparison.

**Video 1:** TTE (parasternal long axis view) showing severely dilated LV, hypokinesis along the septal, inferior, and lateral walls of LV, and depressed EF estimated at <15%. EF, ejection fraction; LV, left ventricle; TTE, transthoracic echocardiography

**Video 2:** TTE (parasternal short axis at the level of the mitral valve) showing enlarged LV with poor contractility and mitral valve with normal coaptation of leaflets. LV, left ventricle; TTE, transthoracic echocardiography

**Video 3:** TTE (apical four-chamber view) showing severely dilated LV, poor contractility of ventricular walls, diffuse hypokinesis of LV, and estimated EF. EF, ejection fraction; LV, left ventricle; TTE, transthoracic echocardiography

The patient was diagnosed with decompensated non-ischemic cardiomyopathy stage C and New York Heart Association (NYHA) class III > IV, likely from use of anabolic steroids. Due to young age, no evidence of hyperlipidemia, and lack of family history of coronary artery disease, no cardiac catheterization was performed to evaluate an ischemic cause of cardiomyopathy, as it was thought to be unlikely. While hospitalized, the patient was initiated on diuretic therapy along with guideline-directed management therapy (GDMT) with an angiotensin-converting enzyme (ACE) inhibitor for afterload reduction, hydralazine, Isordil, and a beta-blocker that was dose-reduced as clinical symptoms improved. The patient denied taking diuretic medications in the past. On follow-up in clinic a week after discharge, the patient presented with mostly compensated heart failure (NYHA class I > II) with much improved dyspnea. He reported cessation of his anabolic steroids and reported feeling no side effects from discontinuation of steroids.

## Discussion

With the growing number of people turning to AASs as a means to boost physique and athletic prowess, the deleterious side effects of these drugs have slowly become uncovered. Exogenous AAS use is rapidly becoming an emergent public health concern, as some estimates indicate that 3-4 million Americans use some form of AAS [6]. AASs have been studied to show interactions within the bone, brain, endocrine, muscle, prostate, hematopoietic, and even CVD system [7]. Anabolic steroids have been linked to various cardiac adverse effects. Some of these effects include myocardial infarction, sudden cardiac death, cardiac tamponade, development of dilated cardiomyopathy, and various arrhythmias such as ventricular fibrillation and atrial fibrillation [1]. Medical literature is now surfacing describing the association between AAS and the CVD system, with some recent studies discussing the association between androgenic steroid use and left ventricular systolic and diastolic dysfunction.

In their observational study on the cardiotoxicity of AAS, Baggish et al. showed that AAS users had significantly reduced LV function compared to non-users (mean EF ± SD: 52%±11 vs 63%±8; p-value < 0.05) as well as more pronounced diastolic dysfunction [4]. Furthermore, their study demonstrated that AAS consumers had considerably more LV hypertrophy than those who did not use, possibly suggesting an anabolic effect on cardiac muscle mass [4]. Whether or not intermittent or cumulative use of AAS leads to these cardiac complications is still very much misunderstood. Some analyses indicate that there is no significant connection between sustained AAS use and cardiac dysfunction, likening AAS cardiotoxicity to that of alcohol-induced cardiotoxicity, where cardiac dysfunction may only be partially and unpredictably related to lifetime usage [5,8]. On the other hand, in a review of 21 articles that showed a link between AAS and ventricular dysfunction and cardiomegaly, the majority of studies found that it was due to long-term use [9].

Although AAS use has clinically shown to result in cardiac dysfunction, the pathophysiology behind the association continues to be a topic of debate. AASs have several interactions with metabolic breakdown pathways and androgenic receptor interactions that have been studied to show a connection to various cardiac pathologies. Stimulation of androgen receptors, found on cardiac myocytes, is theorized to cause structural changes to the myocardium, such as hypertrophy and dilation, resulting in impaired cardiac contraction and relaxation [10]. As a consequence of these androgen receptor effects on the cardiac myocytes, cardiomyopathy, ventricular hypertrophy, and cardiac muscle dilation can result in significant clinical findings and long-term morbidities [11]. Furthermore, there has been evidence of androgenic pathways in the development of cardiac hypertrophy in hearts of both humans and mice. Examples include raised 5α reductase, aromatase, and androgen receptor expression in hypertrophic hearts [12]. Another purported mechanism has to do with the aldosterone-like and growth-promoting effects of AASs on the cardiac muscle, which are followed by apoptosis and rapid influx of intracellular calcium, leading to myocardial fibrosis and ultimately cardiac dysfunction [3,13].

Knowing that ASS-induced cardiac changes can lead to cardiomyopathy and heart failure, it becomes crucial to understand how to manage these complications. Management of AAS-induced cardiomyopathy is similar to that of other non-ischemic causes with the use of GDMT. In heart failure with reduced EF, beta-blockers, ACE inhibitors/angiotensin receptor blockers are first-line agents, as they decrease morbidity and mortality by reversing LV dilation. Spironolactone is generally used when EF is <35%, as prior studies have shown mortality improvement through inhibition of the renin-angiotensin-aldosterone system. Drugs such as ivabradine, hydralazine, and isosorbide dinitrate can be added based on underlying comorbidities and patient characteristics. Our patient showed vast improvement in symptoms with the use of GDMT.

## Conclusions

Our case highlights an interesting presentation of a young male with acute decompensated heart failure with dilated cardiomyopathy secondary to chronic anabolic steroid use. AAS abuse can have critically significant consequences such as impaired cardiac function at an early age, making recognition of the problem and educating users of the effects of AAS crucial in preventing catastrophic health outcomes. Definitive management of AAS-induced cardiomyopathy involves stopping the offending agent and providing appropriate guideline-directed heart failure medications, which can potentially slow and even reverse disease progression, for which some evidence exists. An endocrinologist’s opinion should be sought for the management of AAS withdrawal symptoms. Steroid-induced cardiomyopathy is still poorly understood, and more research needs to be conducted to better understand its complications and long-term effects.

## References

[1] Achar S, Rostamian A, Narayan SM (2010). Cardiac and metabolic effects of anabolic-androgenic steroid abuse on lipids, blood pressure, left ventricular dimensions, and rhythm. Am J Cardiol.

[2] Vorona E, Nieschlag E (2018). Adverse effects of doping with anabolic androgenic steroids in competitive athletics, recreational sports and bodybuilding. Minerva endocrinol.

[3] Maravelias C, Dona A, Stefanidou M, Spiliopoulou C (2005). Adverse effects of anabolic steroids in athletes: a constant threat. Toxicol Lett.

[4] Baggish AL, Weiner RB, Kanayama G (2017). Cardiovascular toxicity of illicit anabolic-androgenic steroid use. Circulation.

[5] Baggish AL, Weiner RB, Kanayama G, Hudson JI, Picard MH, Hutter AM Jr, Pope HG Jr (2010). Long-term anabolic-androgenic steroid use is associated with left ventricular dysfunction. Circ Heart Fail.

[6] Pope HG Jr, Wood RI, Rogol A, Nyberg F, Bowers L, Bhasin S (2014). Adverse health con- sequences of performance-enhancing drugs: an endocrine society scientific statement. Endocr Rev.

[7] Ha Ha, ET ET, Weinrauch ML, Brensilver J (2018). Non-ischemic cardiomyopathy secondary to left ventricular hypertrophy due to long-term anabolic-androgenic steroid use in a former Olympic athlete. Cureus.

[8] Kupari M, Koskinen P, Suokas A (1991). Left ventricular size, mass and function in relation to the duration and quantity of heavy drinking in alcoholics. Am J Cardiol.

[9] Perry JC, Schuetz TM, Memon MD, Faiz S, Cancarevic I (2020). Anabolic steroids and cardiovascular outcomes: the controversy. Cureus.

[10] Joseph J, Yassen Naqvi S, Strum E (2017). Reversible anabolic androgenic steroid-induced cardiomyopathy. Cardiovasc Disord Med.

[11] Han H, Farouque O, Hare DL (2015). Steroid‐induced cardiomyopathy. Med J Aust.

[12] Payne JR, Kotwinski PJ, Montgomery HE (2004). Cardiac effects of anabolic steroids. Heart.

[13] Shamloul RM, Aborayah AF, Hashad A, Abd-Allah F (2014). Anabolic steroids abuse-induced cardiomyopathy and ischaemic stroke in a young male patient. BMJ Case Rep.

